# P-605. Bivalent RSV Prefusion F-Based Subunit Vaccine Generates High and Durable Neutralizing Titers Across an Entire RSV Season among Older Adults

**DOI:** 10.1093/ofid/ofae631.803

**Published:** 2025-01-29

**Authors:** Jose F Cardona, Tarek Mikati, Yasushi Fukushima, Qin Jiang, Daniel P Eiras, John Woodside, Michael Patton, Kumar Ilangovan, Elena Kalinina, David Cooper, Kena A Swanson, Annaliesa S Anderson, Alejandra C Gurtman, Iona Munjal

**Affiliations:** Indago Research & Health Center, Hialeah, Florida; 3. Pfizer, Inc., Vaccine Research & Development, Pearl River, New York; Fukuwa clinic, Chuo-ku, Tokyo, Japan; Pfizer, Collegeville, Pennsylvania; Pfizer, Inc., Pearl River, New York; Pfizer, Collegeville, Pennsylvania; Pfizer, Vaccine Research and Development, Hurley, England, United Kingdom; Vaccine Research and Development, Pfizer, USA, Raleigh, North Carolina; Pfizer, Collegeville, Pennsylvania; Pfizer, Collegeville, Pennsylvania; Pfizer, Collegeville, Pennsylvania; Pfizer, Collegeville, Pennsylvania; Pfizer, Collegeville, Pennsylvania; Pfizer Inc

## Abstract

**Background:**

Pfizer’s bivalent respiratory syncytial virus prefusion F subunit vaccine (RSVpreF [ABRYSVO™]) was approved in the United States for prevention of lower respiratory tract infections (LRTI) caused by RSV in individuals ≥60 years. The objective of this analysis is assessing the durability of RSVpreF vaccination on immunologic response over time.

**Figure 1.**
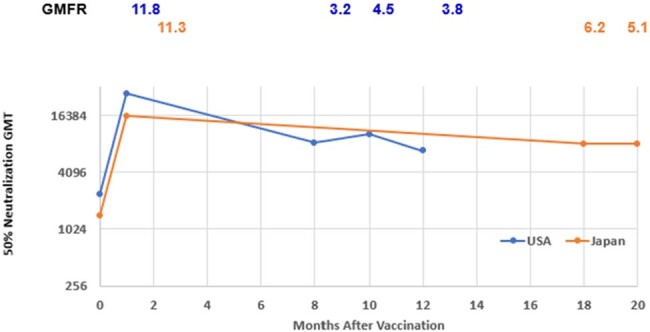
RSV A GMTs and GMFRs by Time Interval After Vaccination with RSVpreF, by Country- Evaluable Immunogenicity Population GMT= geometric mean titer; GMFR= geometric mean fold rise

**Methods:**

The bivalent RSVpreF Efficacy Study was a phase 3, global, randomized, double-blind, placebo-controlled study evaluating VE for RSV-LRTI in adults ≥60 years over two RSV seasons (NCT05035212). A total of 36862 participants received RSVpreF or placebo (1:1) starting in August 2021. VE for LRTI-RSV with 3 or more symptoms was 88.9% (53.6, 98.7) in Season 1 and 77.8% (51.4, 91.1) in Season 2. The immunogenicity subset from selected sites included 1150 participants enrolled in the United States and Japan. Blood samples were collected at baseline prior to vaccination, 1 month post vaccination, and planned prior to the start of the second RSV season. Based on follow-up timelines, serology was draw in US participants (N=307) 8-12 months after vaccination and in Japanese participants (N=230) 18-20 months post vaccination.

**Figure 2.**
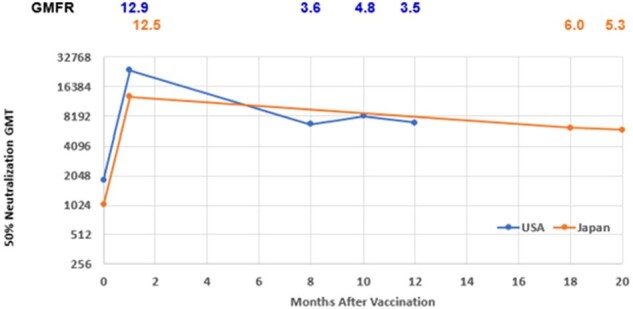
RSV B GMTs and GMFRs by Time Interval After Vaccination with RSVpreF, by Country- Evaluable Immunogenicity Population GMT= geometric mean titer; GMFR= geometric mean fold rise

**Results:**

Overall, RSV GMTs increased substantially after vaccination with RSVpreF (GMFR was 11.6 and 12.7 at 1-month postvaccination for RSV A and B respectively) and remained high at pre-Season 2 (GMFR was 4.7 and 4.8); while remained the same as baseline for placebo group (GMFR ranged from 1.1 to 1.3 for both RSV A and RSV B at both timepoints). When further analyzing data at pre-Season 2 timepoint for RSVpreF, both RSV A (figure 1) and RSV B (figure 2), GMTs remained persistently elevated well above pre-vaccination levels 8-12 months post vaccination among US participants (RSV A GMFR range: 3.2-4.5; RSV B GMFR range: 3.5-4.8) and 18-20 months among Japanese participants (RSV A GMFR range: 5.1-6.2; RSV B GMFR range: 5.3-6.0).

**Conclusion:**

Vaccination with RSVpreF elicited a strong immune response in adults ≥60 years to both RSV A and B and remained durable throughout the first RSV season up to 18-20 months after vaccination, during a time when high VE was demonstrated. This analysis provides further data to support the importance of neutralizing titers in the VE of RSVpreF.

**Disclosures:**

**Tarek Mikati, MD,MPH**, Pfizer, Inc.: salary|Pfizer, Inc.: Stocks/Bonds (Public Company) **Qin Jiang, PhD**, Pfizer: Salary|Pfizer: Stocks/Bonds (Public Company) **Daniel P. Eiras, MD, MPH**, Pfizer. Inc: Employee|Pfizer. Inc: Stocks/Bonds (Private Company) **John Woodside, PhD**, Pfizer: Stocks/Bonds (Public Company)|Pfizer, Inc.: salary|Pfizer, Inc.: Stocks/Bonds (Public Company) **Michael Patton, B.Sc.**, Pfizer: Employee|Pfizer: Stocks/Bonds (Public Company) **Kumar Ilangovan, MD, MSPH, MMCi**, Pfizer, Inc.: salary|Pfizer, Inc.: Stocks/Bonds (Public Company) **Elena Kalinina, PhD**, Pfizer, Inc.: Salary|Pfizer, Inc.: Stocks/Bonds (Public Company) **David Cooper, PhD**, Pfizer, Inc.: Employee|Pfizer, Inc.: Stocks/Bonds (Public Company) **Kena A. Swanson, Ph.D.**, Pfizer: Employee of Pfizer|Pfizer: Stocks/Bonds (Public Company) **Annaliesa S. Anderson, PhD**, Pfizer, Inc.: Employee|Pfizer, Inc.: Stocks/Bonds (Public Company) **Alejandra C. Gurtman, M.D.**, Pfizer, Inc.: Employee|Pfizer, Inc.: Stocks/Bonds (Public Company) **Iona Munjal, MD**, Pfizer: Salaried employee|Pfizer: Stocks/Bonds (Public Company)

